# Dissociation of disease onset, progression and sex differences from androgen receptor levels in a mouse model of amyotrophic lateral sclerosis

**DOI:** 10.1038/s41598-021-88415-0

**Published:** 2021-04-29

**Authors:** Doris Tomas, Victoria M. McLeod, Mathew D. F. Chiam, Nayomi Wanniarachchillage, Wah C. Boon, Bradley J. Turner

**Affiliations:** 1 Florey Institute of Neuroscience and Mental Health, University of Melbourne, 30 Royal Parade, Parkville, VIC 3052 Australia; 2 Perron Institute for Neurological and Translational Science, Queen Elizabeth Medical Centre, Nedlands, WA 6150 Australia

**Keywords:** Neuroscience, Diseases of the nervous system

## Abstract

Amyotrophic lateral sclerosis (ALS) is an adult-onset neurodegenerative disorder caused by loss of motor neurons. ALS incidence is skewed towards males with typically earlier age of onset and limb site of onset. The androgen receptor (AR) is the major mediator of androgen effects in the body and is present extensively throughout the central nervous system, including motor neurons. Mutations in the AR gene lead to selective lower motor neuron degeneration in male spinal bulbar muscular atrophy (SBMA) patients, emphasising the importance of AR in maintaining motor neuron health and survival. To evaluate a potential role of AR in onset and progression of ALS, we generated SOD1^G93A^ mice with either neural AR deletion or global human AR overexpression. Using a Cre-LoxP conditional gene knockout strategy, we report that neural deletion of AR has minimal impact on the disease course in SOD1^G93A^ male mice. This outcome was potentially confounded by the metabolically disrupted Nestin-Cre phenotype, which likely conferred the profound lifespan extension observed in the SOD1^G93A^ double transgenic male mice. In addition, overexpression of human AR produced no benefit to disease onset and progression in SOD1^G93A^ mice. In conclusion, the disease course of SOD1^G93A^ mice is independent of AR expression levels, implicating other mechanisms involved in mediating the sex differences in ALS. Our findings using Nestin-Cre mice, which show an inherent metabolic phenotype, led us to hypothesise that targeting hypermetabolism associated with ALS may be a more potent modulator of disease, than AR in this mouse model.

## Introduction

Amyotrophic lateral sclerosis (ALS) is a fatal progressive neurodegenerative disorder in which upper and lower motor neurons die, leading to muscle atrophy and ultimately respiratory failure^1^. ALS shows increased incidence, a younger age and predominantly limb onset in males^2–4^. Higher incidence of ALS has been identified among professional athletes and soccer players^5–8^ which could be linked to higher prenatal testosterone exposure^9–11^. The biological actions of androgens are mediated by the androgen receptor (AR), a nuclear steroid hormone receptor expressed within tissues throughout the body including the central nervous system (CNS)^12^. The adult onset neurodegenerative disorder, spinal bulbar muscular atrophy (SBMA), is caused by trinucleotide repeat expansions in the *AR* gene, resulting in selective lower motor neuron degeneration in males^13^. This evidence supports a pivotal role for androgens and AR in motor neuron health.

Animal models of ALS reflect the sex differences observed clinically^14,15^. The mutant superoxide dismutase 1 (SOD1^G93A^) mouse is the most well characterised and commonly used model of ALS^16^. Studies into the mechanisms by which sex influences ALS have to date, largely focused on manipulation of hormones. Firstly, castration in male SOD1^G93A^ mice does not modify disease course^17,18^. Several confounds to castration exist. It may lead to the disruption of other hormone sources and steroidogenesis pathways, such as progesterone and estrogens. Alternatively, the adrenal glands provide another source of androgens^19^. Blockade of AR by the anti-androgen, flutamide, exacerbated muscle atrophy and induced an earlier disease onset in male SOD1^G93A^ mice^20^. Administration of the androgen, dihydrotestosterone (DHT), was neuroprotective in SOD1^G93A^ male mice^21^, while the anabolic steroid nandrolone was detrimental^22^. These studies highlight the differential actions and tissue-selectivity of biological and synthetic androgens. Lastly, evidence supports prenatal testosterone exposure potentially having an early impact on motor neuron vulnerability in later life^9^, whereas most of the studies conducted in SOD1^G93A^ mice manipulate AR signalling systems from adulthood at a time when disease onset occurs.

Here, we have used genetic manipulation of AR to study its impact throughout the lifespan of SOD1^G93A^ mice. Despite the compelling evidence for androgen influences in ALS, neither selective neural deletion nor global overexpression of AR modified the disease course of male SOD1^G93A^ mice. The Cre-LoxP conditional knockout strategy we employed to selectively delete AR from neurons and glia, used the Nestin-Cre (NesCre) mouse which inherently displays an altered metabolic phenotype. When crossed with SOD1^G93A^ mice, NesCre inadvertently delayed disease onset and extended survival, revealing that targeting hypermetabolism associated with ALS may be a more effective modulator of disease than sex hormones in this mouse model.

## Results

### Androgen receptor is efficiently and selectively deleted in spinal cord of nARKO mice

**Figure 1:**
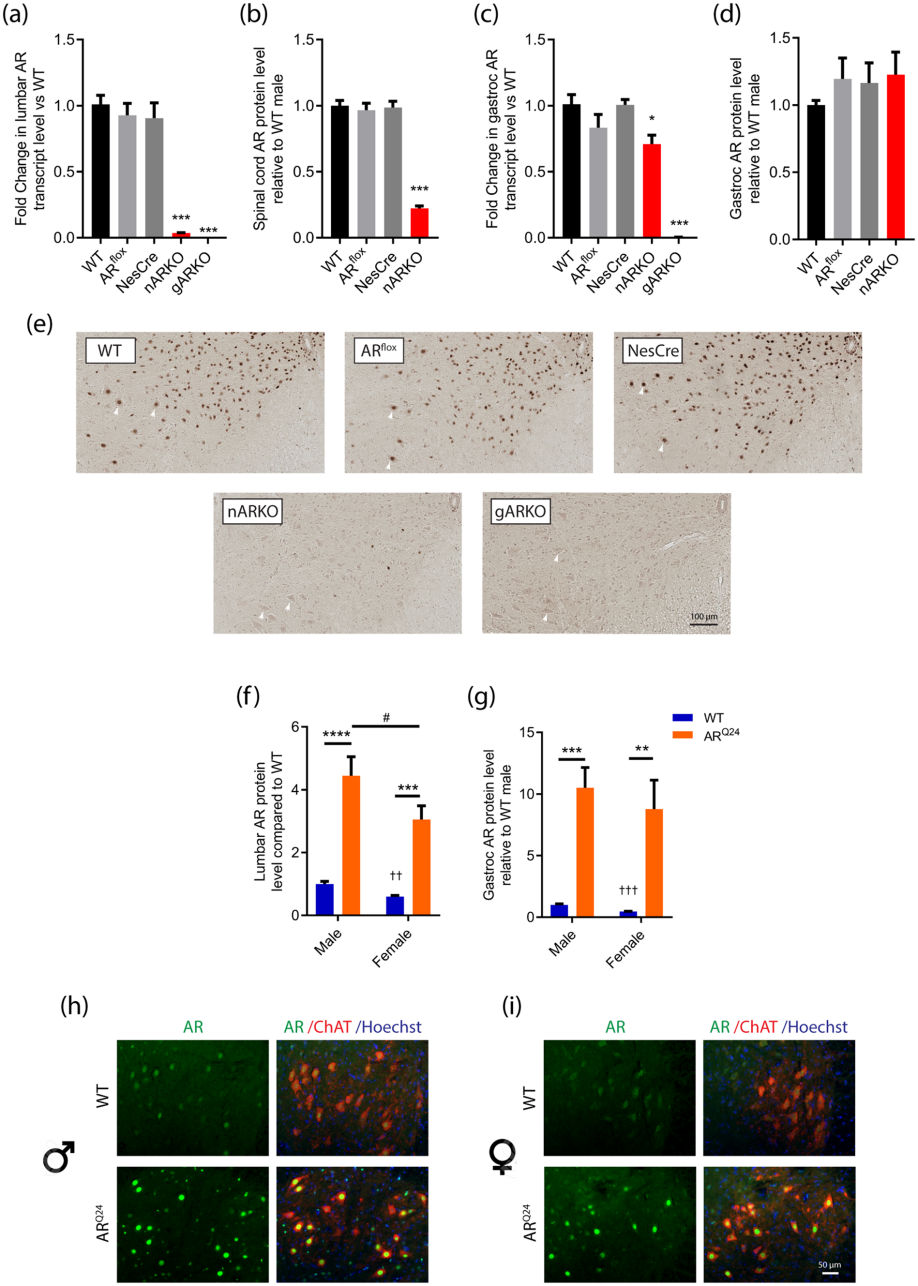
Validation of AR expression in spinal cord and skeletal muscle of nARKO and AR^Q24^ mice. **(a)** AR transcript and **(b)** protein levels in nARKO spinal cord, compared to control genotypes. **(c)** AR transcript and **(d)** protein levels in nARKO gastrocnemius muscle, compared to control genotypes at 2 months age. Data represents mean ± SEM, n = 5 mice per group. * P < 0.05, *** P < 0.001, **** P < 0.0001 by one-way ANOVA with Dunnett’s multiple comparison test comparing all genotypes to WT. Full-length blots are presented in Supplementary Fig. 1a,b. **(e)** AR immunohistochemical staining in the ventral horn spinal cord of nARKO mice, compared with control genotypes, at 6 months age. Representative motor neurons indicated by white arrowheads. **(f)** AR protein expression in the lumbar spinal cord and **(g)** gastrocnemius muscle of male and female AR^Q24^ transgenic mice, relative to male WT levels at 3 months age. Data represents mean ± SEM, n = 6 mice per group. **P < 0.01, ***P < 0.001, ****P < 0.0001 by two-way ANOVA with Sidak's multiple comparisons test comparing genotype effect; ^#^P < 0.05 by two-way ANOVA with Sidak's multiple comparisons test comparing sex effect. ^††^P < 0.01, ^†††^P < 0.001 compared to WT male by unpaired t-test. Full-length blots are presented in Supplementary Fig. S1c,d. **(h)** AR immunostaining in the lumbar spinal cord of male and **(i)** female WT and AR^Q24^ mice. Motor neurons are identified by positive ChAT staining.

**Figure 2:**
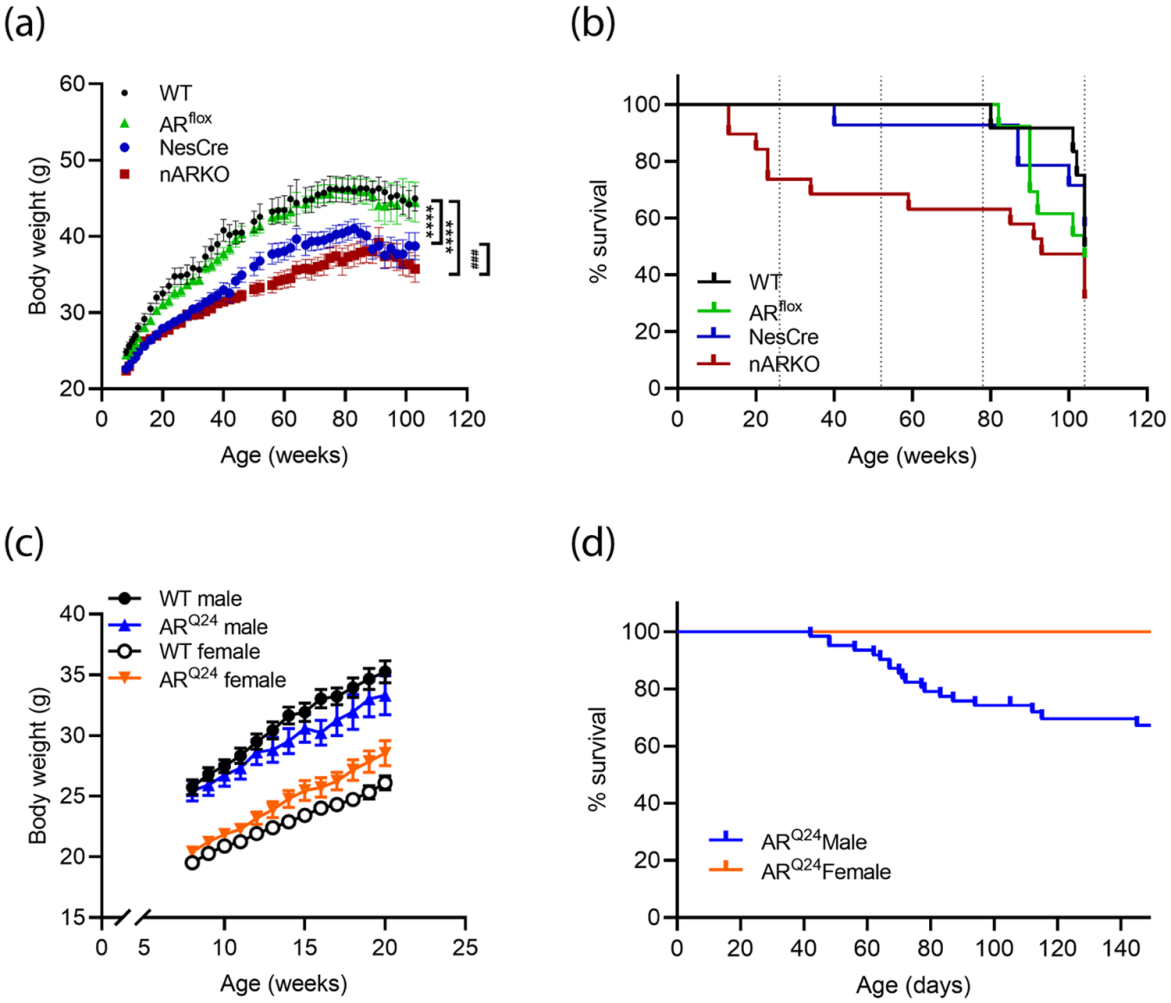
Impact of neural AR deletion and global AR overexpression on male mouse survival. **(a)** Body weight analysis of AR^flox^ x NesCre cross mice over 24 months. Mean ± SEM, n = 11–13 mice per group. **** P < 0.0001 vs WT; ^###^ P < 0.001 vs NesCre by mixed-effects analysis with Tukey’s multiple comparison test for main genotype effect. **(b)** Kaplan–Meier curves of AR^flox^ x NesCre cross mouse survival over 24 months; n = 12 WT; n = 13 AR^flox^; n = 14 NesCre; n = 10 nARKO. **(c)** Body weight analysis of WT and AR^Q24^ mice to P150. Mean ± SEM, n = 12–14 mice per group. (d) Kaplan–Meier curves of AR^Q24^ transgenic mouse survival over 150 days; n = 62 males.

We first used a Cre-LoxP system in mice to obtain CNS-conditional deletion of AR. The NesCre transgenic expresses Cre-recombinase under the rat nestin promotor and enhancer, resulting in AR deletion from neuronal and glial precursor cells^23^. AR transcript was abolished by 96% of wild-type (WT) levels in neural AR knockout (nARKO) spinal cord (Fig. 1a) with a corresponding 78% depletion of AR protein (Fig. 1b, S1a). Gastrocnemius muscle revealed a 30% reduction in AR transcript (Fig. 1c) with no change in AR protein level detected in nARKO mice (Fig. 1d, S1b). In lumbar spinal cord, nuclear AR was not detected in ventral horn motor neurons of nARKO mice which was comparable to the global knockout (gARKO) mouse (Fig. 1e). All 3 genotypes WT, AR^flox^ and Nes-Cre expressed AR abundantly in nuclei of motor neurons in the spinal cord (Fig. 1e). Organ weight analysis showed consistently increased spleen (0.061 ± 0.007 g vs. 0.078 ± 0.014 g; *P* = 0.035) and seminal vesicle (0.12 ± 0.035 g vs. 0.24 ± 0.053 g; *P* < 0.0001) weights in nARKO mice compared to NesCre controls, and reduced testis weights (0.16 ± 0.012 g vs. 0.12 ± 0.009 g *P* = 0.0002) from 2 months age onwards (Table S1). Increased seminal vesicle weights reflect elevated testosterone levels, a commonly observed concomitant of neuronal AR deletion models due to disruption of the hypothalamic-pituitary–gonadal (HPG) axis^24,25^. The AR^flox^ mouse line, used in the current studies, was modified to remove a neomycin resistance cassette^26^ which interfered in AR gene function in several previous AR floxed models^27,28^. There is no known observable interference from loxP insertions in this mouse, confirmed by our assessment of long-term growth curves, major organ weights and AR quantification being indistinguishable from WT mice (Fig. 1a, 2a, S1, Table S1).

### AR is robustly overexpressed in motor neurons and skeletal muscle of AR^Q24^ male and female mice

The AR^Q24^ mouse expresses 5 copies of the WT human AR transgene driven by the chicken β-actin promoter^29^. In lumbar spinal cord, total AR protein levels were increased 4.4-fold in male AR^Q24^ mice, compared to WT controls, while in females this was slightly lower at threefold increase (Fig. 1f, S1c). In gastrocnemius muscle, total AR protein level was elevated 10.5-fold in male AR^Q24^ mice, compared to WT controls (Fig. 1g, S1d). In AR^Q24 ^females, AR was increased 8.8-fold compared to WT male mice. Male vs. female WT levels were not significantly different using two-way ANOVA analysis due to the dominating effect of the AR^Q24^ levels. When analysed separately, female WT mice had 40% less AR in lumbar spinal cord compared to males and 54% less AR in gastrocnemius, consistent with our previous study^20^. AR protein overexpression was reflected in ventral horn spinal cord immunohistochemistry (Fig. 1h,i), where nuclear AR staining was substantially greater in ChAT^+^ motor neurons of AR^Q24^ male and female spinal cord, as well as appearing in the smaller nuclei of surrounding cell populations. Organ weights were comparable between male AR^Q24^ and WT controls, except for decreased skeletal muscle weight observed in AR^Q24^ mice (Table S2).

### Neural AR deletion and global AR overexpression have a detrimental effect on survival of male mice

Male mice carrying the NesCre transgene displayed significant growth retardation over 24 months, compared to WT males, while AR^flox^ males displayed no overt impairments in growth and body weight (Fig. 2a). This has been reported previously in NesCre transgenic mice which have mild hypopituitarism and growth hormone deficiency^30^. Body weight growth was further reduced in nARKO mice, compared to NesCre, revealing a requirement of AR for weight gain. Mice reached maximal weights of 49 ± 5.1 g, 49 ± 5.5 g, 43 ± 4.8 g and 39 ± 6.0 g for WT, AR^flox^, NesCre and nARKO male mice, respectively. At 6 months of age, little difference in organ weights were observed between NesCre and nARKO mice, other than increased prostate weight (0.08 ± 0.01 g vs. 0.13 ± 0.05 g; *P* = 0.0132) and previously mentioned spleen, seminal vesicle and testis differences (Table S1). Off-target AR deletion was evident in kidney, skeletal muscle and heart tissue in 6-month old nARKO mice (Fig S2), and elevated liver AR transcript and protein levels were apparent through secondary mechanisms with no genomic deletion of AR evident (Fig S2). Spontaneous deaths occurred in nARKO mice from 13 weeks (3 months) of age onward, with 8/10 mice showing no signs of ill-health prior to death (Fig. 2b). In this study cohort, 6/19 mice reached 24 months of age without appreciable weight loss, adverse events or evidence of abnormal gross pathology. Control genotypes began losing mice beyond 18 months of age, typically due to general poor health and weight decline. Mice reaching 24 months of age with no evidence of poor health included 6/12, 6/13 and 8/14 mice for WT, AR^flox^ and NesCre groups, respectively (Fig. 2b). Aged nARKO mice showed significant enlargement of the prostate up to 6-fold, compared to NesCre controls (0.108 ± 0.016 g vs. 0.681 ± 0.179 g; *P* < 0.0001 for nARKO and NesCre mice, respectively. Table S1). No other differences in gross organ pathology were notable.

We analysed the effects of AR overexpression in male and female mice (Fig. 2c). A slight downward weight trend in AR^Q24^ males and upward trend in AR^Q24^ females was observed, compared to WT counterparts. This genotype effect was only found to be significant in females when analysed by repeated measures two-way ANOVA (F_(1,22)_ = 5.056; *P* = 0.0349). Unexpectedly, sudden deaths were observed in approximately one third of male AR^Q24^ mice by 150 days (Fig. 2d). These mice frequently showed a sudden rapid weight gain prior to death, indicative of fluid retention. Cardiac hypertrophy was evident on post-mortem inspection and was confirmed as the cause of death by external veterinary pathology. The occurrence of these deaths appear to coincide with rising circulating testosterone levels which we have previously reported in mice from P60-P150 age^31^. The heart weight of remaining healthy AR^Q24^ mice did not show a difference, compared to control WT mice, indicating incomplete penetrance of this phenotype in AR^Q24^ male mice (Supplementary material Fig S3a). Previously, this genotype was backcrossed onto the C57BL/6 J genetic background and spontaneous deaths were not described in the phenotype^29^. Notably, the current B6D2 mixed background exhibits greater heart weight than C57BL/6 J counterparts (0.22 ± 0.03 g vs. 0.12 ± 0.01 g; Fig S3), hence AR effects on cardiac phenotype may be exacerbated in mice on the B6D2 background. Likewise, nARKO heart weights were not different to NesCre controls throughout their lifespan (Fig S3b).

### Neural deletion of AR does not alter disease course in SOD1^G93A^ male mice

**Figure 3:**
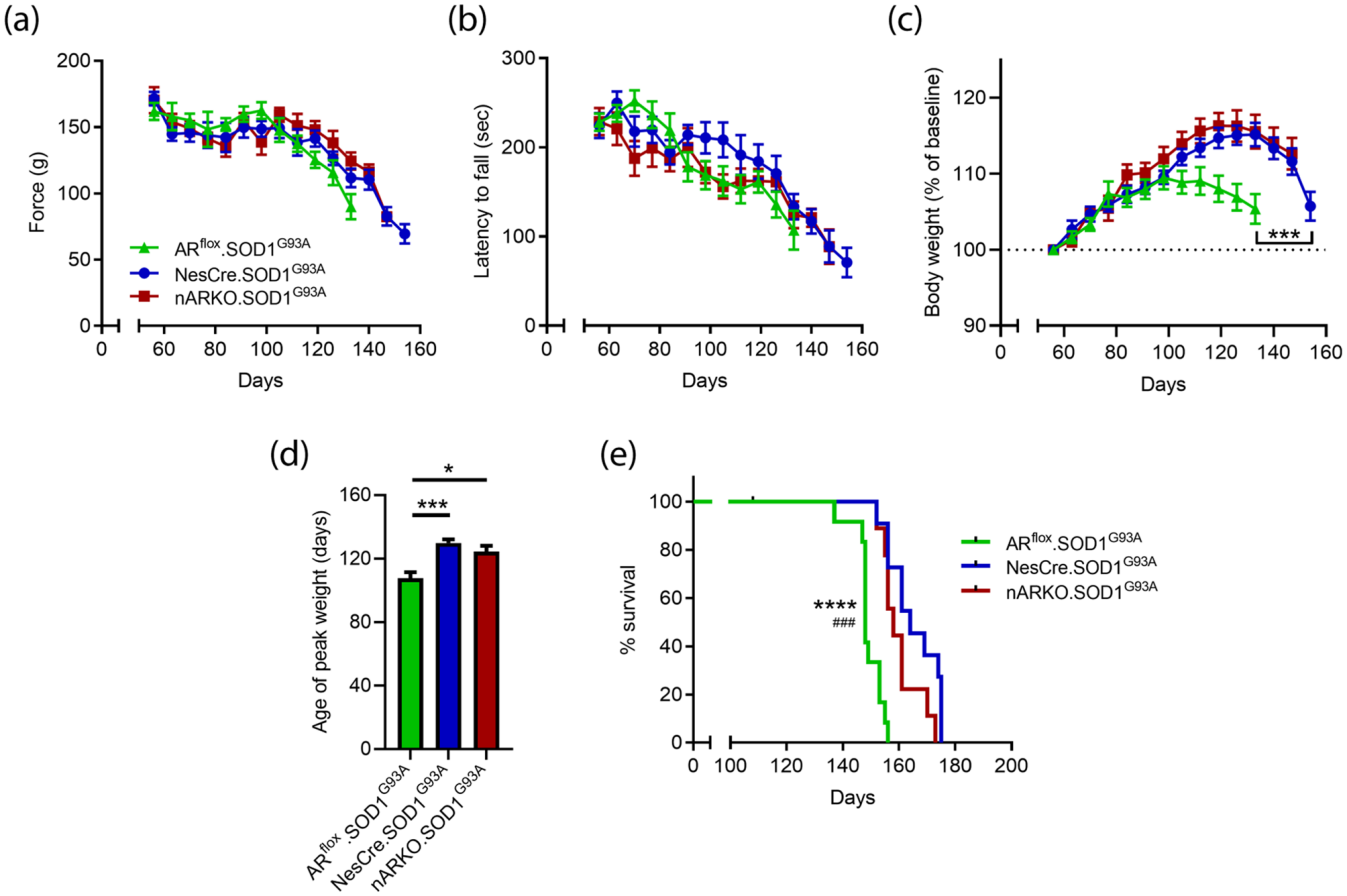
Neural deletion of AR does not alter disease course in SOD1^G93A^ male mice. Motor function assessment by **(a)** four limb grip strength and **(b)** rotarod performance. **(c)** Body weight analysis of AR^flox^ x NesCre x SOD1^G93A^ cross mice over disease course; ***P < 0.001 vs. NesCre:SOD1^G93A^ by two-way ANOVA with Tukey’s multiple comparison test comparing main genotype effect. **(d)** Age in weeks when peak weight occurred; P < 0.001 by one-way ANOVA with Dunnett’s multiple comparison test comparing all genotypes to NesCre:SOD1^G93A^. **(e)** Kaplan–Meier curves show reduced survival in AR^flox^:SOD1^G93A^ compared to NesCre:SOD1^G93A^ (****P < 0.0001) and nARKO:SOD1^G93A^ (^###^P < 0.001). All values presented as mean ± SEM; n = 9–12 mice per group.

After establishing the effects of AR ablation in non-diseased mice, we next determined the impact of AR ablation in the CNS, on the phenotype of male transgenic SOD1^G93A^ mice. SOD1^G93A^ mice were generated with nARKO, AR^flox^ or NesCre transgenes. A comparison of limb muscle strength (Fig. 3a) and locomotor function (Fig. 3b) revealed no overt genotype differences. Body weight analysis showed an earlier symptomatic decline in the AR^flox^:SOD1^G93A^ genotype from 105 days onwards (*P* = 0.0002; Fig. 3c). Age of peak body weight was used to retrospectively assess disease onset (Fig. 3d). Onset was 108 ± 13 days in the AR^flox^:SOD1^G93A^ group, in line with a previous recent assessment conducted on identical SOD1^G93A^ mice from within our laboratory, reporting onset at 110 ± 20 days using this method^20^. NesCre:SOD1^G93A^ mice showed a delay in disease onset by 22 days, compared to AR^flox^:SOD1^G93A^ (*P* < 0.0001) reaching peak body weight at 130 ± 8 days. nARKO:SOD1^G93A^ disease onset was slightly earlier than NesCre:SOD1^G93A^ mice at 124 ± 11 days, although this 6-day difference was not statistically significant (*P* = 0.4623). NesCre:SOD1^G93A^ and nARKO:SOD1^G93A^ mice showed a 16 and 10 day median survival extension, respectively, compared to AR^flox^:SOD1^G93A^ (Fig. 3e). nARKO:SOD1^G93A^ mice showed a modest reduction in median survival by 6 days, compared to NesCre:SOD1^G93A^ which approached statistical significance (*P* = 0.07) by the log-rank test. Taken together, the NesCre phenotype appears to be driving the survival extension in SOD1^G93A^ male mice.

### Overexpression of AR does not influence disease parameters in SOD1^G93A^ mice

**Figure 4:**
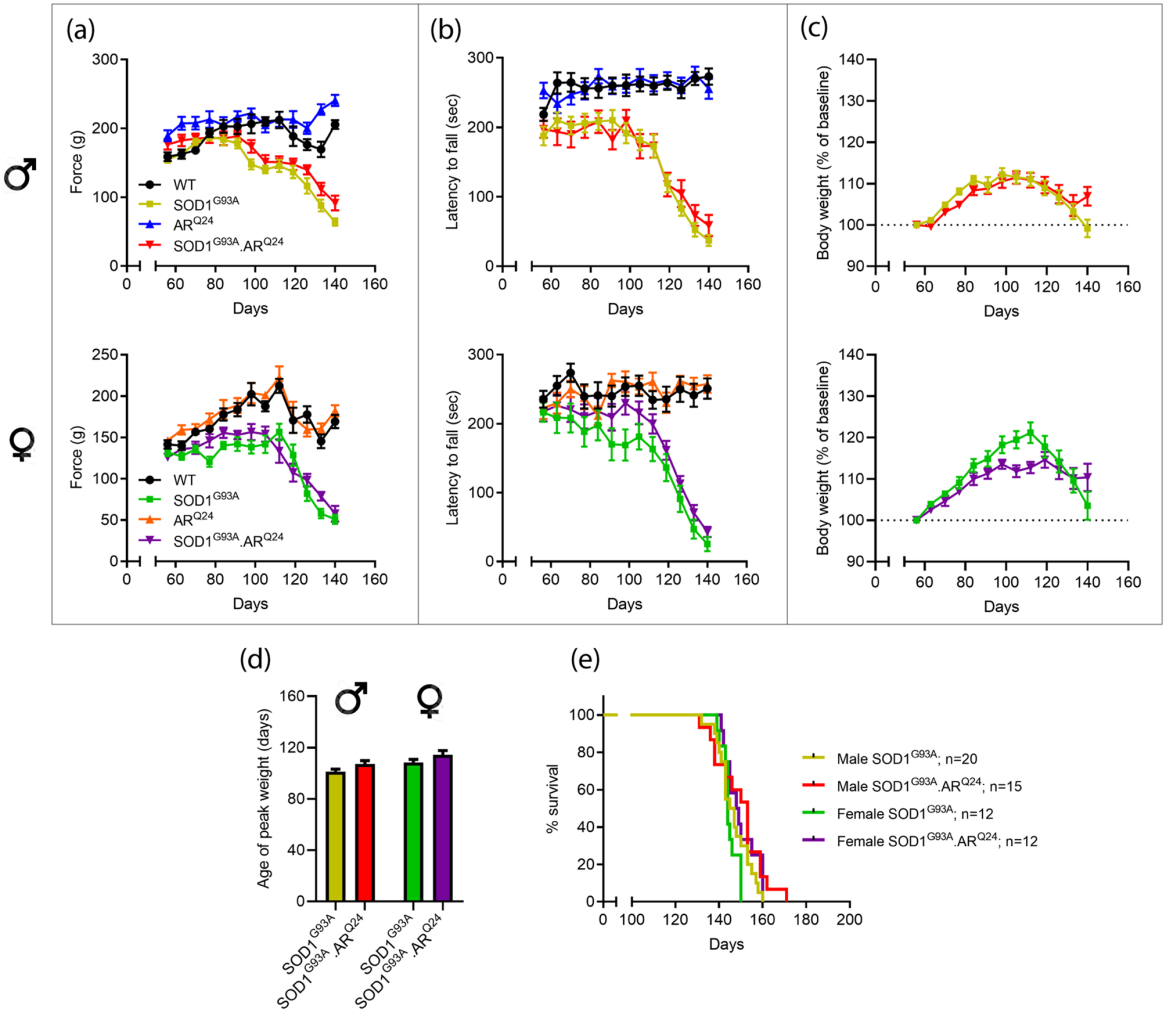
Global overexpression of AR does not influence disease progression and survival in SOD1^G93A^ mice. Motor function assessment by **(a)** four limb grip strength and **(b) **rotarod performance and **(c)** body weight analysis of AR^Q24^ x SOD1^G93A^ cross male and female mice over disease course. **(d)** Age in weeks when peak weight occurred in males and female. **(e)** Kaplan–Meier curves showing comparable survival in SOD1^G93A^:AR^Q24^ mice compared to SOD1^G93A^ mice. All values presented as mean ± SEM; n = 12–20 mice per group.

With evidence that neither deletion of AR from neurons, nor peripheral AR antagonism^20^ are capable of modifying disease outcome in SOD1^G93A^ mice we turned to an overexpression model. The effects of global AR overexpression were evaluated in SOD1^G93A^ mice. SOD1^G93A^ mice were crossed with AR^Q24^ mice, giving rise to WT, SOD1^G93A^, AR^Q24^ and SOD1^G93A^:AR^Q24^ genotypes. Muscle strength showed comparable decline in SOD1^G93A^ and SOD1^G93A^:AR^Q24^ mice with consistent performance maintained in aged-matched non-disease counterparts, AR^Q24^ and WT mice (Fig. 4a). In SOD1^G93A^ male mice, the onset of decline in grip strength was 89 ± 9 days compared to 103 ± 12 days in female mice (P = 0.0006 by unpaired t-test, Fig S4a). AR overexpression did not have a significant effect on the onset of decline in grip strength in SOD1^G93A^ mice, with onset occurring at 95 ± 10 days and 102 ± 11 days in SOD1^G93A^:AR^Q24^ male and female mice, respectively. Similar trends were observed in locomotor performance (Fig. 4b) with the onset of decline in function occurring at 102 ± 10 days compared to 110 ± 7 days in male and female SOD1^G93A^ mice, respectively (P = 0.0221 by unpaired t-test, Fig S4b). Again, AR overexpression did not have an effect on disease onset determined by locomotor performance, occurring at 107 ± 9 days and 106 ± 9 days in SOD1^G93A^:AR^Q24^ male and female mice, respectively. Comparable weight declines were apparent between SOD1^G93A^ and SOD1^G93A^:AR^Q24^ genotypes (Fig. 4c). Age of peak body weight showed a significant effect of sex (F_(1,56)_ = 7.82; *P* = 0.0071) and genotype (F_(1,56)_ = 5.24; *P* = 0.0258) on disease onset when analysed by two-way ANOVA (Fig. 4d). Multiple comparisons did not reveal significant intergroup differences. Separate analysis of male vs. female SOD1^G93A^ mice again revealed males had a significantly earlier disease onset compared to female mice (P = 0.0271 by unpaired t-test, Fig S4c), showing a consistent trend across multiple methods of determining disease onset in SOD1^G93A^ mice. The median lifespan of male SOD1^G93A^ mice (146 days) was comparable to SOD1^G93A^:AR^Q24^ mice (153 days) (*P* = 0.1494). Likewise, in females, no survival differences were apparent, with median survival of 144 vs. 149 days for SOD1^G93A^ and SOD1^G93A^:AR^Q24^ mice, respectively (*P* = 0.0810; Fig. 4e).

### Changes in AR expression do not drive an alteration in skeletal muscle IGF-1 transcript

**Figure 5:**
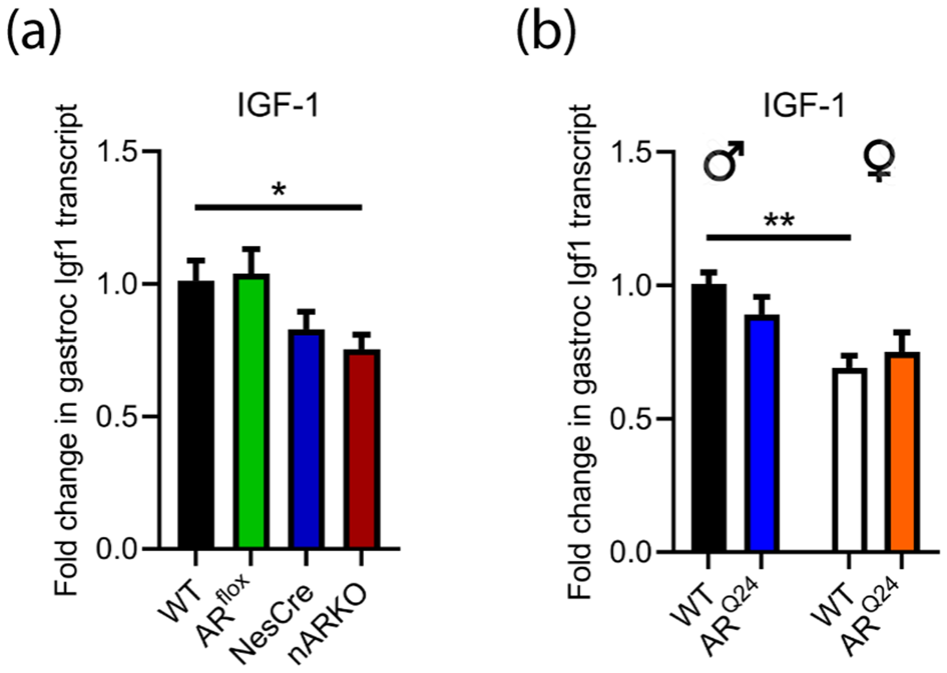
AR expression levels do not drive an alteration in skeletal muscle IGF-1 transcript. **(a)** IGF-1 transcript levels in gastrocnemius muscle of AR^flox^ x NesCre male mice at 2 months age. Data represents mean ± SEM, n = 5 mice per group. *P < 0.05 by one-way ANOVA with Dunnett’s multiple comparison test comparing all genotypes to WT. **(b)** IGF-1 transcript levels in gastrocnemius muscle of male and female AR^Q24^ mice compared to WT at 3 months age. Data represents mean ± SEM, n = 6 mice per group. **P < 0.01 by two-way ANOVA with Sidak's multiple comparisons test comparing sex effect.

Insulin-like growth factor 1 (IGF-1) is a known transcriptional target of skeletal muscle AR^32^ and mediates local trophic effects^33^ and neuroprotective actions in motor neurons^34^. IGF-1 expression in muscle was not altered in AR^flox^ mice, whereas it appears to be downregulated in the NesCre mice by 17% compared to WT (Fig. 5a). While nARKO mice were significantly downregulated by 25% compared to WT males, there is no difference when compared to NesCre controls (Fig. 5a). Interestingly, there was no effect of overexpressing AR on muscle IGF-1 expression (Fig. 5b). A sex effect is observed (F_(1,56)_ = 5.24; *P* = 0.0258) with female WT mice showing a 30% reduction in IGF-1 transcript compared to WT males, indicating that androgen levels rather than AR levels, dictate regulation of IGF-1.

## Discussion

We have explored the impact of genetic manipulation of AR on motor performance, growth and survival in healthy and diseased mice. To selectively delete AR within the CNS, we crossed the floxed AR mouse with the NesCre mouse which expresses Cre-recombinase in neural progenitor cells. Neural AR deletion did not show significant impacts upon disease progression and survival of SOD1^G93A^ transgenic mice compared to NesCre controls. The introduction of the NesCre transgene to SOD1^G93A^ mice profoundly increased survival by 16 days, compared to those carrying the phenotypic benign AR^flox^ transgene. Overexpression of AR did not modify disease progression and survival in SOD1^G93A^ male or female mice. However, both AR deletion and overexpression models negatively influenced the survival of non-diseased male mice with incomplete penetrance.

The widespread presence of AR and systemic testosterone action throughout the body make it difficult to isolate tissue-specific impacts of AR signalling. Deletion of AR from the CNS results in disruptions to the HPG axis regulating testosterone release, resulting in elevated circulating testosterone levels^25,35^. In a calcium/calmodulin-dependent protein kinase IIα (CaMKIIα)-iCre driven ARKO mouse, testosterone levels were increased 2-fold and seminal vesicle weight increased 4-fold^25^. In nARKO mice, 2 to 4-fold elevations in circulating testosterone have been reported^24,35^, reflecting the ~ 2-fold increased seminal vesicle weight in our study. The NesCre driver line also reportedly has activity outside of the intended target, causing further potential confounds^36,37^. Therefore, the implications of altered AR signalling in the periphery of nARKO mice cannot be discounted. In gastrocnemius muscle, we report no net change to AR protein level in nARKO mice, despite a 30% reduction in AR transcript level. This could be due to altered local androgen levels affecting AR protein turnover^31^. In myocyte-specific KO mice, AR was not found to contribute to androgen-mediated hypertrophy in gastrocnemius or other limb muscles, although it negatively impacted muscle strength^38,39^. Conversely, we have previously reported that systemic administration of the AR competitive antagonist, flutamide, promoted muscle atrophy and enhanced disease onset, without altering survival in SOD1^G93^^A^ male mice^20^. Interestingly, restricted overexpression of WT AR in muscle had a detrimental effect, with mice developing a neurodegenerative phenotype similar to SBMA models^40^. Our global overexpression model, which expresses human WT AR, decreased gastrocnemius muscle weight, mildly reduced body weight in non-disease males, and had no influence on disease progression in SOD1^G93A^ mice. The reason for the discrepancies observed between these overexpressing models could be the different promotors used to drive transgene expression. In the skeletal muscle-specific model, AR^Q22^ expression was driven by α-actin which is far more abundant in muscle compared to β-actin^41^, used to drive global AR expression in the current study. It is possible that the level of AR in muscle in the former model prompted a toxic gain of function. In models of SBMA it has been shown that expression of the polyglutamine expanded AR in muscle alone, is capable of driving neurological components of the disease. Males have an earlier disease onset in both mouse models and clinical ALS, with denervation and muscle atrophy among the earliest pre-symptomatic pathologies of the disease^42^. Further exploration of the role of skeletal muscle AR in driving disease onset in ALS models is warranted.

The perturbation of AR signalling outside of the CNS in these models is likely responsible for the negative survival effects observed in non-disease models. This is especially relevant for the AR^Q24^ overexpression model where sudden death appeared to be the result of cardiac hypertrophy. The role of androgens in human cardiovascular disease is contradictory. The risk of cardiovascular disease is higher in males than females, which could indicate a negative impact of AR signalling. Likewise, androgens acting via AR impair recovery of cardiac function after ischemia–reperfusion injury^43^ and promote chronic kidney disease^44^. Conversely, lower testosterone levels in ageing males correlates with greater cardiovascular disease mortality^45^. In global ARKO models, mice exhibit increased atherosclerosis, higher circulating cholesterol and increased development of cardiac fibrosis, as reviewed by Chang and colleagues^46^. In nARKO mice, we observed genetic deletion of AR within both kidney and heart tissue. This age-dependence and contradictory role of AR in cardiovascular disease is reflected in our survival data in AR genetic models. Deaths in nARKO mice, typically occurred beyond 5 months of age; in AR^Q24^ mice this occurred much earlier coinciding with rising circulating testosterone levels.

We have previously reported that AR is depleted in the lumbar spinal cords of male SOD1^G93A^ mice^31^. In light of this finding, further abolishment of AR signalling, through AR deletion, may have limited impact on disease course and survival. We did observe a mild 6-day reduction in median survival of nARKO:SOD1^G93A^ males, compared to NesCre:SOD1^G93A^ males, although not significant. A higher prenatal testosterone exposure associates with ALS^9^. This contrasts to evidence that early postnatal testosterone administration is neuroprotective against cortical injury in adult rodents^47^. In NesCre mice, Cre-LoxP recombination is reported to have occurred throughout the CNS by E15.5^36^. This is prior to the first testosterone surge, occurring between E17-19 in rodents^48^. Therefore, we anticipate that the direct effects of early prenatal testosterone exposure on neurons is sufficiently abolished in nARKO:SOD1^G93A^ mice and has minimal impact on adult disease course. The levels of testosterone in AR^Q24^ mice have not been reported. No change in testosterone levels were evident in a CMV-promoter driven AR overexpression model^49^. It therefore seems unlikely that dysregulation in testosterone production and reduced AR signalling is responsible for the lack of effect observed in the SOD1^G93A^:AR^Q24^ model. Quantification of skeletal muscle IGF-1 transcript, a known AR transcriptional target, was not altered by AR overexpression. A lower level of the transcript was observed in female WT mice, implicating local androgens as driving transcript levels independent of AR level. This is supported by evidence that exogenous administration of DHT to male SOD1^G93A^ mice, significantly upregulated IGF-1 transcript in tibialis anterior muscle^21^.

In the current study we did not observe AR-mediated alteration in disease onset or outcome in SOD1^G93A^ mice. While we cannot rule out that model limitations confounded the ability to detect an underlying role for AR, it is also possible that other factors are mediating the sex-specific differences in ALS. Estrogen receptors (ER) are abundantly expressed by motor neurons and all glial cell populations in the lumbar spinal cord of SOD1^G93A^ mice^31^. Exogenous estradiol has potent anti-inflammatory actions and provides neuroprotection to motor neurons when administered to male SOD1^G93A^ mice^50^. Exogenous progesterone, stimulated autophagy in the spinal cord of male SOD1^G93A^ mice, delaying disease onset and extending survival^51^. Ovariectomy in female SOD1^G93A^ mice did not impact disease onset, but accelerated disease progression^52^. Together, these studies present a strong case for estrogens and progesterone having multiple neuroprotective and disease modifying actions in SOD1^G93A^ mice. Alternatively, it is possible that sex chromosome effects could be mediating sex differences in ALS, independently of gonadal hormones^53^. For example, in multiple sclerosis and autoimmune disease, differential methylation of the X-chromosome gene, Foxp3, has been identified in T lymphocytes; possibly resulting from X-chromosome imprinting^54,55^. In Parkinson’s disease, the expression of Y chromosome gene, SRY, is upregulated and promotes nigrostriatal degeneration^56^. This may contribute to the male bias of the disease.

The NesCre mouse has been described as having a metabolic phenotype^37^ caused by the ectopic expression of the human growth hormone gene in the hypothalamus^57^. This causes a decrease in growth hormone regulating hormone (GHRH) release by mouse hypothalamic neurosecretory neurons. In turn this leads to a 70–80% reduction in growth hormone (GH) release by the anterior pituitary^30^. Likewise, the pituitary hormones prolactin (PRLH) and thyroid stimulating hormone (TSH) were similarly decreased. GH is responsible for promoting growth, hence, the observed impairment in weight gain in the NesCre mice. GH operates to stimulate lipid and carbohydrate metabolism acting primarily on the adipose/fat tissue and liver. We have summarised the disrupted energy metabolism effects described for NesCre transgenic mice in Fig S5. Notably, a 50% reduction in liver IGF-1 transcript, a major target of GH action, was observed^57^. Most of these actions, including increased leptin production, increased insulin sensitivity and higher adiposity^58^, act to promote energy storage. Additionally, the reduced TSH production by the pituitary and resulting thyroid hormone deficiency would be expected to contribute to a hypometabolic state^59^ in the NesCre mouse.

Hypermetabolism, defined by increased resting energy expenditure, is a well described early phenomenon in ALS patients. It does not appear to correlate with age, sex or BMI^60^, although clearly associates with poor prognosis^61^. Likewise, hypermetabolism is also an early pre-symptomatic feature of disease in SOD1^G93A^ mice^62–65^. The mechanisms leading to hypermetabolism in the SOD1^G93A^ mice appear to be highly complex and have only recently begun to be unravelled. A GH deficiency and decrease in IGF-1 signalling were evident in advanced symptomatic SOD1^G93A^, reflecting clinical ALS^62^. Further studies revealed GH secretions fluctuate with disease course showing an elevation coinciding with onset of disease^65^. The authors suggested this to be a response to denervation, in an attempt to stimulate muscle repair through IGF-1 upregulation. We show decreased IGF-1 transcript in skeletal muscle of NesCre mice, therefore, this pathway unlikely presents the mechanism of neuroprotection when crossed with SOD1^G93A^ mice. Skeletal muscle plays a major role in determining systemic metabolic rate^66^ and seems a likely contributor to metabolic dysfunction in ALS. In SOD1^G93A^ mice an increase in fatty acid oxidation occurs in muscle, reflecting a switch toward the used of lipids as a primary fuel source in peripheral tissues^64,67^. In NesCre mice, increased lipid uptake and storage occurs and may counteract the dysregulation in energy source occurring in SOD1^G93A^ mice (Fig S5). This is supported by evidence of lifespan extension in SOD1^G93A^ mice when crossed with a transgenic harbouring a Dynein *Cra* mutation; the latter causing defective lipolysis and increased lipid stores^68^. The resulting double transgenic mice showed an increased adiposity and restoration in the use of carbohydrate energy sources. More recently, impaired glucose homeostasis was shown in symptomatic SOD1^G93A^ mice^69^. This was characterised by two major defects: increased glucose uptake, independent of altered insulin sensitivity; and impaired glucagon signalling, the pancreatic hormone stimulating conversion of liver glycogen to glucose. NesCre mice have been shown to have dysregulated insulin and glucose sensitivity, presenting another potential mechanism to counter SOD1^G93A^ hypermetabolism.

In conclusion, we demonstrate here that genetic perturbations to AR levels in mice can have detrimental impacts on male mice, although, these do not exacerbate or alter the disease course in SOD1^G93A^ mice. Furthermore, AR manipulation alone may be insufficient to modulate disease with circulating and local tissue hormone levels likely to be the driving force behind sex differences in ALS. Finally, the altered metabolism in NesCre mice has inadvertently provided striking evidence that combating early hypermetabolism in SOD1^G93A^ is likely a key target in modulation of ALS progression.

## Methods

### Animals

All experiments on mice were conducted in accordance with the Australian National Health and Medical Research Council published Code of Practice and approved by the Florey Institute of Neuroscience and Mental Health Animal Ethics Committee (approval numbers 16-001-FINMH and 17-074-FINMH). The study was carried out in compliance with the ARRIVE guidelines. Transgenic SOD1^G93A^ mice (B6.Cg-Tg(SOD1*G93A)1Gur/J line; stock number 004435), AR^flox^ mice^26^ (B6.129S1-Ar^tm2.1Reb^/J line, stock number 018450) and Nestin-Cre (NesCre) mice^23^ (B6.Cg-Tg(Nes-cre)1Kln/J line, stock number 003771) were purchased from the Jackson Laboratory (Bar Harbor, ME, USA) were maintained on a C57BL/6J background. Transgenic AR^Q24^ mice^29^ (B6D2-Tg(CAG-AR*24Q)5-5Sobu; stock number RBRC00827) were purchased from RIKEN BioResource Centre and maintained on a B6D2 background.

To generate conditional neural ARKO mice, heterozygous female AR^fl/wt^ mice were crossed with male NesCre^+/-^ to generate four litter-matched male genotypes; non-transgenic WT, AR^fl^, NesCre^+/-^ and AR^fl^:NesCre^+/-^ (nARKO). To generate constitutive ARKO mice for negative control tissue, AR^fl/wt^:NesCre^+/-^ double heterozygous transgenic females were bred with male mice giving rise to AR null males (gARKO) through germline recombination, as previously described^70^. Given the high frequency of germline recombination in female AR^fl/wt^:NesCre^+/-^ breeders^70^ it was not viable to pair with SOD1^G93A^ male breeders. Male SOD1^G93A^ mice were crossed with female NesCre^+/-^ to generate male SOD1^G93A^:NesCre^+/-^ transgenic mice. Due to SOD1^G93A^ and NesCre transgenes being located on chromosome 12, in male SOD1^G93A^:NesCre^+/-^, these two transgenes were segregated during meiosis and not inherited together in F1 progeny. We acquired a unique transgenic male SOD1^G93A^:NesCre^+/-^ through chance breeding whereby the SOD1^G93A^ and NesCre^+/-^ became linked to chromosome 12 through homologous recombination. These mice were crossed with AR^fl/wt^ heterozygous females to produce AR^wt^:NesCre^+/-^:SOD1^G93A^ (NesCre:SOD1^G93A^) and AR^fl^: NesCre^+/-^:SOD1^G93A^ triple transgenic male (nARKO:SOD1^G93A^) littermates. A parallel breeding strategy was set up where SOD1^G93A^ mice were crossed with AR^fl/wt^ heterozygous females to generate AR^fl^:SOD1^G93A^ males (AR^flox^:SOD1^G93A^).

To generate AR overexpressing SOD1^G93A^ mice, female AR^Q24^ heterozygous mice were crossed with male SOD1^G93A^ to generate four litter-matched male genotypes; non-transgenic WT, SOD1^G93A^, AR^Q24^ and SOD1^G93A^:AR^Q24^. The mice were a mixed isogenic F1 background.

Animals were group-housed under standard 12 h light–dark conditions with access to standard rodent chow and water. At 2, 3 or 6 months of age, animals were killed by lethal dose of sodium pentobarbitone (100 mg/kg, i.p.) and organs collected, weighed and snap frozen in dry-ice. For immunohistochemistry, mice were cardiac perfused with 0.1 M PBS followed by 4% paraformaldehyde, sucrose cryoprotected and snap freezing in isopentane as previously described^31^.

### RT-qPCR

Spinal cord and gastrocnemius muscle were prepared and RNA extractions performed as previously described^20,31^. Brain, liver, kidney, gastrocnemius and heart tissue from NesCre and nARKO mice were mechanically pulverised using liquid nitrogen prior to processing for DNA, RNA and protein extraction. Genomic DNA (gDNA) was extracted from tissue samples overnight using PureLink Genomic DNA Mini Kit (Invitrogen) according to manufacturer’s protocol. Primer sequences for gDNA were: *Ar*^*ex1*^ forward 5′- AAG CAG GTA GCT CTG GGA CA -3′, *Ar*^*ex1*^ reverse 5′- GAG CCA GCG GAA AGT TGT AG -3′; and internal control *DAG1* forward 5′- CCA AGG AGC AGA TCA TAG GGC -3′, *DAG1* reverse 5′- AGA GCA TTG GAG AAG GCA GG -3′. For RNA extraction tissue was homogenised in QIAzol Lysis Reagent (Qiagen, Cat# 79306) using the TissueLyserLT (Qiagen) for 5 min at 50 Hz and RNA containing phase isolated using chloroform prior to further purification using RNeasy Mini Kit following manufacturer’s protocol. Primer sequences for RNA were: *Ar* forward 5′- GTG AAA TGG GAC CTT GGA TG -3′, *Ar* reverse 5′- GCC AGA AGC TTC ATC TCC AC -3′; *Igf1* forward 5′- TGG ATG CTC TTC AGT TCG TG -3′, *Igf1* reverse 5′- GCA ACA CTC ATC CAC AAT GC -3′; and internal control *Hprt1* forward 5′- GAT CAG TCA ACG GGG GAC AT -3′, *Hprt1* reverse 5′- CAT TTT GGG GCT GTA CTG CTT -3′. RT-qPCR was performed as previously described^20^ and samples analysed in triplicate with Ct values normalized to the housekeeping gene. Fold change between WT control and transgenic groups was determined using the 2^−ΔΔCt^ method.

### Western blotting

Spinal cord and gastrocnemius muscle were prepared and protein extractions performed as previously described^20,31^. Brain, liver, kidney and heart tissue was homogenised by sonication (50% amplitude pulses applied over 10–15 s) in ice-cold RIPA buffer (50 mM Tris–Cl, pH 7.4, 150 mM NaCl, 0.1% SDS, 1% sodium deoxycholate and 1% Triton-X 100) containing protease and phosphatase inhibitor cocktail tablets. Protein lysates were denatured and electrophoresed through 4–15% Criterion TGX Stain-Free gels (Bio-Rad Laboratories, NSW, Australia) or 4–20% Mini-PROTEAN TGX Stain-Free gels (for brain, liver, kidney and heart supernatants) and transferred onto PVDF membrane using a Trans-Blot Turbo Transfer System (Bio-Rad) as previously described^20,31^. Blots were probed overnight at 4 °C with rabbit primary antibody against AR (1:1000, Abcam, cat# ab133273, RRID:AB_11156085) in SignalBoost Immunoreaction Enhancer (Merck Millipore, Cat# 407207) followed by 1 h room temperature incubation with StarBright Blue 700 goat anti-rabbit secondary antibody (1:5000, Bio-Rad, Cat# 12004161, RRID:AB_2721073). For analysis, background adjusted AR band intensity was normalised to total lane protein intensity using Image Lab 6.0 software (Bio-Rad, www.bio-rad.com/en-au/product/image-lab-software, RRID:SCR_014210). Average group values were then expressed fold relative to averaged WT control group values (expressed as 1.0).

### Immunohistochemistry

Lumbar spinal cord was cryosectioned at 20 µm and slide mounted onto poly-l-lysine coated glass slides. Citrate antigen retrieval was performed as previously described^31^. For fluorescent detection immunostaining was performed as previously described^31^ with the following primary antibodies: rabbit anti-AR (1:200, Abcam, Cat# ab133273) and goat anti-ChAT (1:200, Millipore, Cat# AB144P, RRID:AB_2079751). The following secondary antibodies were incubated for 2 h at room temperature; donkey biotinylated-anti-rabbit (1:200, Jackson ImmunoResearch Cat# 711-065-152, RRID:AB_2340593), streptavidin Alexa Fluor-488 (1:200, Jackson ImmunoResearch Cat# 016-540-084, RRID:AB_2337249) and anti-goat DyLight-550 (1:200, Thermo Fisher Scientific Cat# SA5-10087, RRID:AB_2556667). Hoechst 33342 (Invitrogen) DNA stain was incubated for 15 min at 1 µg/ml. All images were captured on a Zeiss AxioObserver Z1 (Carl Zeiss Pty Ltd, North Ryde, Australia). For chromogenic DAB detection, sections were blocked for 15 min in 0.5% hydrogen peroxide in PBS and 1 h at room temperature in animal-free blocking reagent (Cell Signalling Technology, Cat# 15019). Rabbit anti-AR (as described above) was incubated at 4 °C for 48 h in SignalStain antibody diluent (Cell Signaling Technology, Cat# 8112). SignalStain Boost IHC Detection Reagent (HRP, rabbit) (Cell Signaling Technology, Cat# 8114) was used as secondary detection and DAB colorimetric reaction performed using SignalStain DAB Substrate Kit (Cell Signaling Technology, Cat# 8059). Images were acquired on a Leica DMLB2 microscope.

### Behavioural analyses and survival assessment

For aging of AR^flox^ x NesCre mouse cohorts (n = 12–19 from 2 months age onwards), animals were weighed fortnightly until 24 months of age. Mice reaching endpoint criterion included decline in physical condition and/or progressive non-recoverable weight loss amounting to 20% of peak weight. The survival of male AR^Q24^ mice was determined across a cohort of 62 mice to age 150 days. In SOD1^G93A^ mouse survival studies mice were weighed once weekly and assessed for motor function. Locomotion was assessed using an accelerating rotarod (Rota-Rod 47600, Ugo Basile, Italy) as previously reported^20^. Muscular strength was assessed using a grip strength meter (BIO-G53, Bioseb, US). Mice were suspended by the tail and lowered onto a slanted metal grid until all four paws briefly made contact. Mice were then pulled from the mesh grid in parallel to the attached force transducer in a rapid, smooth and continuous motion. Force was measured in grams and an average of five successive pulls was reported for each mouse. The appearance of disease onset was determined retrospectively using the age of maximal body weight. Clinical endpoint for survival was defined as onset of paralysis in the hindlimbs and/or a cumulative loss of 20% peak body weight. In the AR^flox^ x NesCre x SOD1^G93A^ cohort, n = 9–13 mice per group reached clinical endpoint and were included in the dataset. Once mouse from nARKO group died at 108 days prior to any symptom onset and was included as a censored value. In the AR^Q24^ x SOD1^G93A^ cohort group sizes included in survival data analysis were n = 20 male SOD1^G93A^, n = 15 male SOD1^G93A^:AR^Q24^, n = 12 female SOD1^G93A^ and n = 12 female SOD1^G93A^:AR^Q24^.

### Data analyses and statistics

Transcript, western blot, tissue weight and peak body weight data from AR^flox^ x NesCre cross mice were analysed by one-way ANOVA with Dunnett’s multiple comparison comparing each genotype against WT. Western blot and peak body weight data from AR^Q24^ mice were analysed by two-way ANOVA with Sidak’s multiple comparison test for sex and genotype effects where F-value indicated a significant effect (*P* < 0.05). Cumulative body weight gain plots were analysed by either mixed-effects analysis (for 24 month aged mice) or two-way ANOVA with repeated measures and Tukey’s multiple comparison test for main genotype effect. Survival data was analysed by Log-rank (Mantel-Cox) test. All other single genotype comparisons were performed using two-tailed Student’s t-test. Statistical analysis were performed using GraphPad Prism 8.3 (San Diego, CA, USA) and data presented as mean ± SEM unless otherwise stated.

## Supplementary Information

Supplementary Information.
