# Supplementary material for: Dissociation of disease onset, progression and sex differences from androgen receptor levels in a mouse model of amyotrophic lateral sclerosis
Source: Sci Rep. 2021 Apr 29;11:9255. doi: 10.1038/s41598-021-88415-0 (PMC8085012; doi:10.1038/s41598-021-88415-0)
Supplement: Supplementary file 1 — Supplementary Information. [file 41598_2021_88415_MOESM1_ESM.pdf]

Supplementary Material

Dissociation of disease onset, progression and sex differences from androgen receptor levels in a mouse model of amyotrophic lateral sclerosis

Doris Tomas[1], Victoria M. McLeod[1], Mathew D.F. Chiam[1], Nayomi Wanniarachchillage[1], Wah C. Boon[1], Bradley J. Turner[1,*]

[1]*Florey Institute of Neuroscience and Mental Health, University of Melbourne, Parkville, VIC 3052, Australia.*

Table S1. Tissue weights of AR^{flox}:NesCre transgenic mice

	WT	AR^{flox}	NesCre	nARKO	gARKO
2 months old	(g)	(g)	(g)	(g)	(g)
Liver	1.13 ± 0.20	1.08 ± 0.21	1.03 ± 0.20	1.01 ± 0.17	1.04 ± 0.07
Kidney	0.338 ± 0.030	0.347 ± 0.041	0.261 ± 0.039 **	0.265 ± 0.043 **	0.252 ± 0.021 ***
Heart	0.136 ± 0.017	0.131 ± 0.014	0.119 ± 0.010	0.117 ± 0.027	0.114 ± 0.011
Lung	0.145 ± 0.005	0.145 ± 0.007	0.129 ± 0.014	0.133 ± 0.020	0.156 ± 0.020
Spleen	0.077 ± 0.010	0.081 ± 0.014	0.061 ± 0.007	0.078 ± 0.014 ††	0.096 ± 0.004 * ††††
Pancreas	0.189 ± 0.048	0.195 ± 0.019	0.180 ± 0.029	0.158 ± 0.036	0.121 ± 0.011 **
Gastrocnemius	0.137 ± 0.007	0.134 ± 0.010	0.116 ± 0.010 *	0.105 ± 0.018 ****	0.111 ± 0.007 **
Thymus	0.047 ± 0.006	0.058 ± 0.008 *	0.050 ± 0.006	0.040 ± 0.006	0.086 ± 0.009 ****
Seminal Vesicles	0.143 ± 0.019	0.121 ± 0.044	0.119 ± 0.035	0.237 ± 0.053 *** †††	
Testis	0.176 ± 0.020	0.161 ± 0.021	0.159 ± 0.012	0.117 ± 0.009 **** †††	0.014 ± 0.003 **** ††††
Prostate	0.053 ± 0.007	0.045 ± 0.006	0.045 ± 0.012	0.061 ± 0.015	
6 months old					
Liver			1.34 ± 0.10	1.36 ± 0.15	
Kidney			0.327 ± 0.028	0.338 ± 0.053	
Heart			0.149 ± 0.011	0.162 ± 0.024	
Lung			0.163 ± 0.022	0.170 ± 0.015	
Spleen			0.072 ± 0.007	0.086 ± 0.010 ††	
Pancreas			0.166 ± 0.027	0.151 ± 0.032	
Gastrocnemius			0.147 ± 0.011	0.140 ± 0.007	
Thymus			0.059 ± 0.009	0.052 ± 0.016	
Seminal Vesicles			0.283 ± 0.046	0.731 ± 0.197 ††††	
Testis			0.187 ± 0.019	0.139 ± 0.013 ††††	
Prostate			0.081 ± 0.010	0.129 ± 0.048 †	
24 months old					
Heart	0.200 ± 0.049	0.174 ± 0.023	0.170 ± 0.019	0.143 ± 0.017	
Testis	0.180 ± 0.025	0.182 ± 025	0.180 ± 0.032	0.094 ± 0.019 *** †††	
Prostate	0.100 ± 0.016	0.099 ± 0.020	0.108 ± 0.016	0.681 ± 0.179 **** ††††	

Mean ± SD, n=4-8. * P<0.05, ** P<0.01, *** P<0.001, **** P<0.0001 compared to WT; † P<0.05, †† P<0.01, ††† P<0.001, †††† P<0.0001 compared to NesCre

Table S2. Tissue weights of AR^{Q24} transgenic mice

	WT male	AR^{Q24} male	WT female	AR^{Q24} female
3 months old	(g)	(g)	(g)	(g)
Liver	1.61 ± 0.16	1.63 ± 0.23	1.20 ± 0.14 **	1.20 ± 0.19
Kidney	0.565 ± 0.085	0.518 ± 0.080	0.311 ± 0.037 ****	0.346 ± 0.023
Heart	0.216 ± 0.028	0.218 ± 0.028	0.148 ± 0.020 ***	0.148 ± 0.016
Lung	0.204 ± 0.017	0.299 ± 0.157	0.168 ± 0.014	0.167 ± 0.042
Spleen	0.081 ± 0.019	0.084 ± 0.018	0.073 ± 0.011	0.086 ± 0.016
Pancreas	0.196 ± 0.038	0.170 ± 0.024	0.134 ± 0.025 **	0.167 ± 0.042
Gastrocnemius	0.161 ± 0.022	0.134 ± 0.008 **	0.118 ± 0.010 ****	0.125 ± 0.011
Thymus	0.041 ± 0.010	0.032 ± 0.007	0.056 ± 0.005 **	0.046 ± 0.008
Seminal Vesicles	0.294 ± 0.040	0.316 ± 0.058		
Testis	0.195 ± 0.038	0.205 ± 0.019		
Prostate	0.084 ± 0.023	0.090 ± 0.008		
Ovary			0.018 ± 0.002	0.027 ± 0.003 ###

Mean ± SD, n=6. ** P<0.01, *** P<0.001, ****P<0.0001 compared to WT male; ### P<0.001 compared to WT female

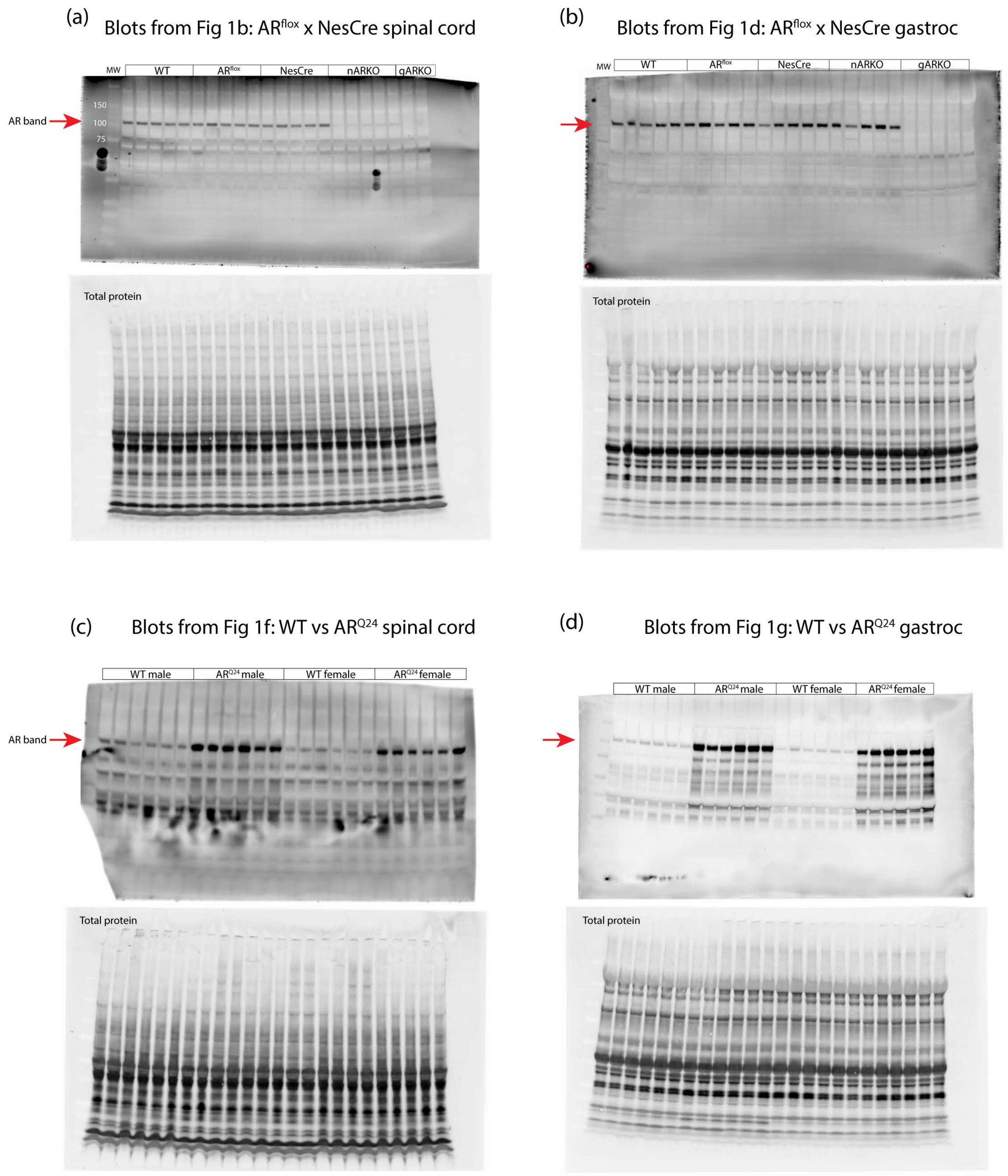

Figure S1. Western blots of spinal cord and gastrocnemius tissue AR (110 kDa) and total protein of data presentened in Figure 1. (a) AR in the spinal cord tissue and (b) gastrocnemius of progeny from AR^{flox} x NesCre crossed mice. (c) AR in the spinal cord and (d) gastrocnemius of overexpressing ARQ24 mice compared to their wildtype counterparts.

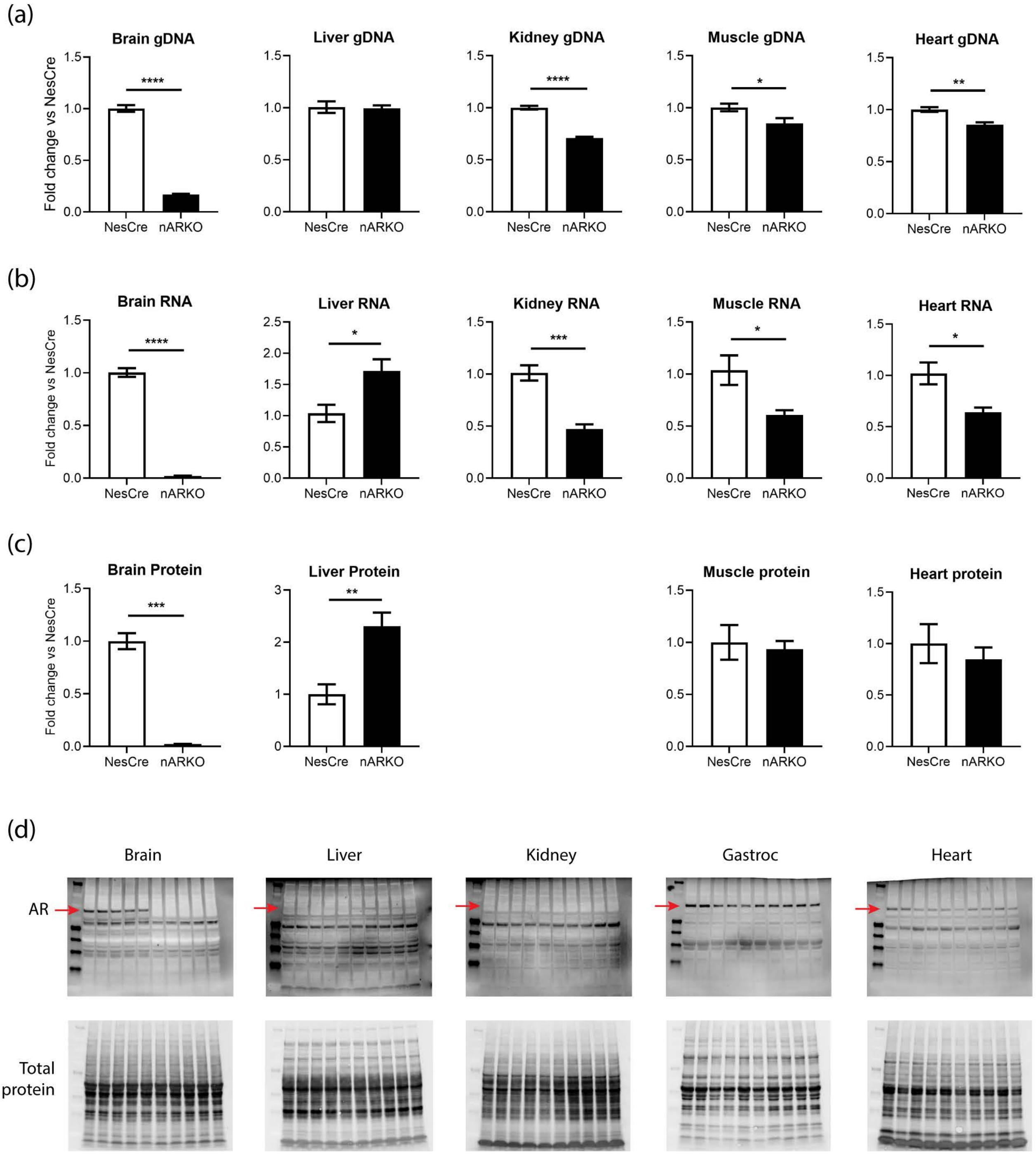

Figure S2. AR deletion in the neuronal ARKO mouse tissues compared to NesCre controls in 6 month old mice at the level of (a) genomic DNA, (b) RNA and (c) protein. (d) Western blots of AR (110 kDa) and total protein in tissues (note accurate levels of AR could not be detected in kidney tissue).

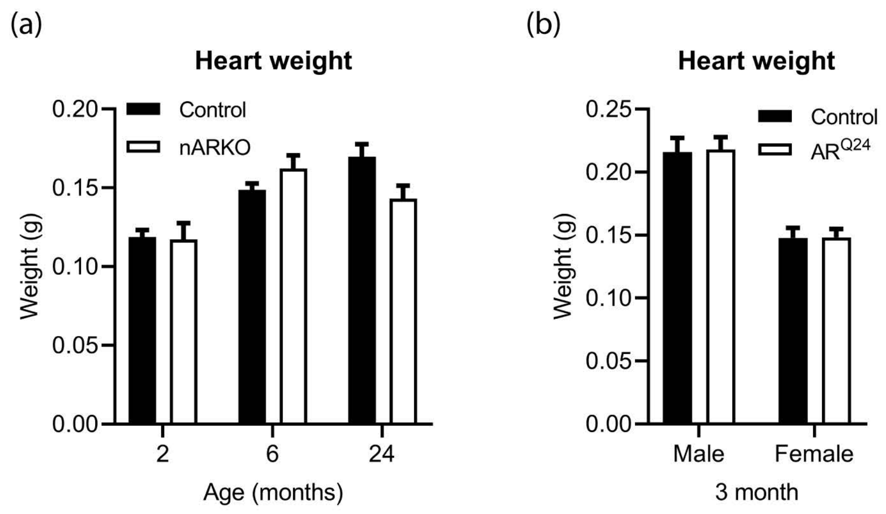

Figure S3. Heart weights of (a) nARKO males at 2, 6 and 24 months age compared to NesCre controls (n=4-8 mice per group) and male and female ARQ24 transgenic mice at 3 months compared to WT controls (n= 6-8 mice per group). Mean ± SEM.

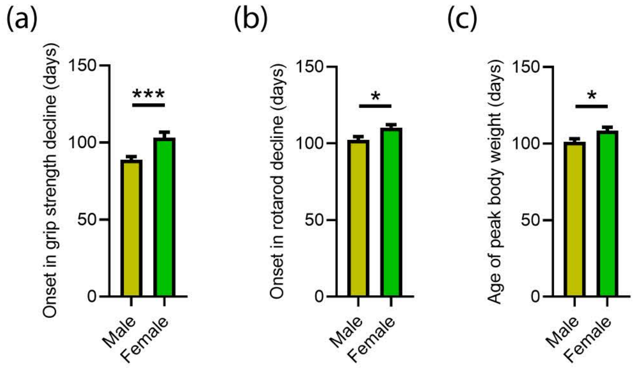

Figure S4. Comparison of disease onset between male and female $SOD1^{G93A}$ mice measured by (a) decline in grip strength, (b) decline in locomotor performance and (c) age of peak body weight. Data represents mean ± SEM (n=≥12). * P<0.05, *** P<0.001 by unpaired t-test.

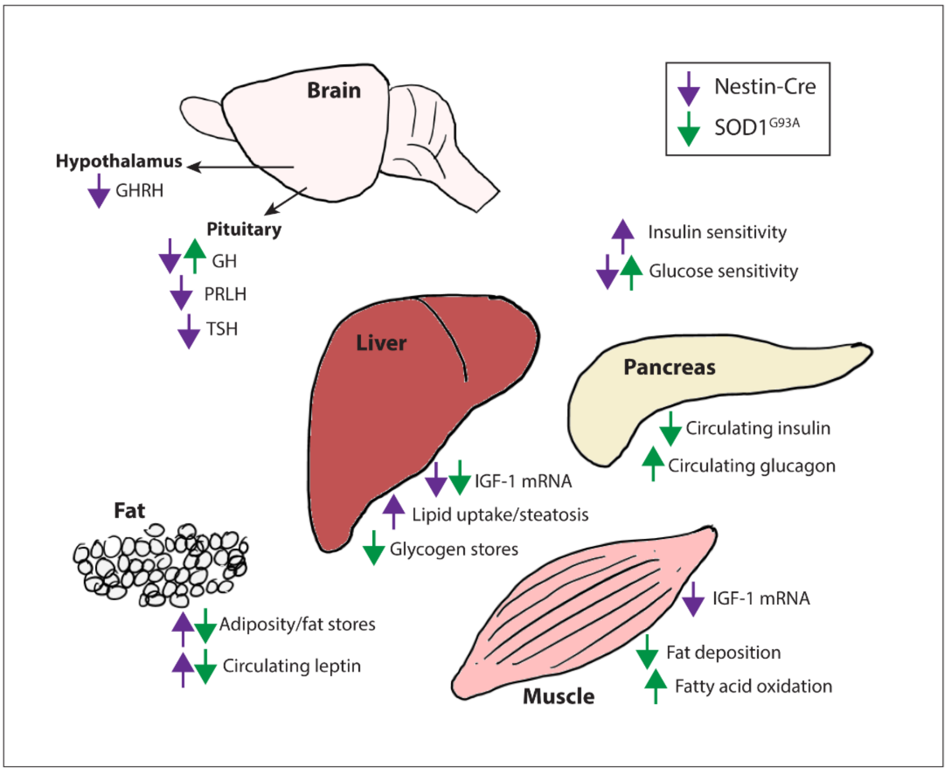

Figure S5. Central and peripheral changes contributing to the altered metabolic condition in Nestin-Cre and SOD1^{G93A} mice. Growth-hormone releasing hormone (GHRH), growth hormone (GH), prolactin releasing hormone (PRLH), thyroid stimulating hormone (TSH).
